# 3-[Bis(pyridin-2-ylmethyl)amino]-5-(4-carboxyphenyl)-BODIPY as Ratiometric Fluorescent Sensor for Cu^2+^

**DOI:** 10.3390/ma11050814

**Published:** 2018-05-16

**Authors:** Akira Hafuka, Hisashi Satoh, Koji Yamada, Masahiro Takahashi, Satoshi Okabe

**Affiliations:** 1 Department of Integrated Science and Engineering for Sustainable Society, Faculty of Science and Engineering, Chuo University, 1-13-27 Kasuga, Bunkyo-ku, Tokyo 112-8551, Japan; 2 Division of Environmental Engineering, Faculty of Engineering, Hokkaido University, North-13, West-8, Sapporo 060-8628, Japan; qsatoh@eng.hokudai.ac.jp (H.S.); m-takaha@eng.hokudai.ac.jp (M.T.); sokabe@eng.hokudai.ac.jp (S.O.); 3 Division of Environmental Materials Science, Graduate School of Environmental Science, Hokkaido University, North-10, West-5, Sapporo 060-0810, Japan; yamada@ees.hokudai.ac.jp

**Keywords:** boron-dipyrromethene, di(2-picolyl)amine, cation, ion, copper, heavy metal, transition metal, dye, fluoroionophore, fluorescence

## Abstract

We developed an asymmetric fluorescent sensor **1** for Cu^2+^, based on 4,4-difluoro-4-bora-3a,4a-diaza-*s*-indacene (BODIPY), by introducing 4-carboxyphenyl and bis(pyridin-2-ylmethyl)amine groups at the 5- and 3-positions, respectively, of the BODIPY core. We then investigated the photophysical and cation-sensing properties of the sensor. BODIPY **1** showed large absorption and fluorescence spectral shifts on binding to Cu^2+^. The fluorescence peak at 580 nm red-shifted to 620 nm. The binding stoichiometry of BODIPY **1** and Cu^2+^ was 1:3. The ratio of the fluorescence intensity at 620 nm to that at 580 nm (*F*_620_/*F*_580_) increased with increasing concentration of Cu^2+^ (3–10 equiv); this enabled ratiometric determination of Cu^2+^. Although BODIPY **1** showed good selectivity for Cu^2+^, there was an interfering effect of Fe^3+^. BODIPY **1** could be used for the naked-eye detection of Cu^2+^ in a water-containing sample.

## 1. Introduction

The conservation of natural environments and their protection from hazardous contaminants are becoming considerably important. Heavy metals, in particular, have serious risks to the environment and to human health because of their toxicity. Copper (Cu) is one of the heavy metals, and excess uptake of Cu into the human body can lead to gastrointestinal disturbance, and liver or kidney damage [1]. The development of rapid and easy screening methods for heavy metals, including Cu, is therefore needed. Presently, the most general analytical methods for heavy-metal determination are inductively coupled plasma (ICP)-optical emission spectrometry, ICP-mass spectrometry, and atomic absorption spectrometry [2]. Although these analytical methods are precise and sensitive, they are costly, and always need complicated sample preparation. Furthermore, they are not used in on-site screening. The development of a cost-effective and easy method for determining concentrations of heavy metals is therefore necessary.

Fluorescence spectroscopy with fluorescent sensor molecules is an attractive method for quantifying heavy-metal cations, because of its high sensitivity, operational simplicity, and versatile instrumentation [3]. In addition, these sensor molecules can determine the free cation concentration of heavy metals, which is important in natural environments and many biological systems. The design and synthesis of new fluorescent sensor molecules are active areas of research, and different kinds of fluorescent sensors for heavy-metal cations have been reported [4]. Among them, ratiometric fluorescent sensors that show fluorescence spectral shift caused by interaction with heavy-metal cations are more sensitive than those that show increase (“turn-on”) or decrease (“turn-off”) of fluorescence intensity [5]. Ratiometric fluorescent sensors enable reliable measurements of analyte concentrations, because the ratio of the intensities at two different fluorescence wavelengths is not affected by fluctuations in the source light intensity and concentration of the fluorescent sensor molecule.

Our strategy for the development of a ratiometric fluorescent sensor for heavy-metal cations is based on the synthesis of an asymmetric 4,4-difluoro-4-bora-3a,4a-diaza-*s*-indacene (BODIPY) fluorescent molecule with a cation receptor at the 3-position. BODIPY has many useful characteristics, such as narrow fluorescence and absorption spectrum, high fluorescence quantum yields, large molar absorption coefficients, and excellent photochemical stability [6]. In addition, BODIPY derivatives can be excited with visible light. The most distinctive characteristic of BODIPY derivatives is that their photophysical properties can be modified by appropriate substitution [7]. Among the eight positions on the BODIPY core, substitution at the 3- and/or 5-position(s) can cause significant shifts in the absorption and fluorescence spectra [7]. The introduction of a cation receptor at the 3- and/or 5- position(s) of BODIPY can therefore be used to create a ratiometric fluorescent sensor [8,9,10,11,12,13]. However, the development of a ratiometric fluorescent sensor for Cu^2+^ is difficult, because Cu^2+^ often shows fluorescence quenching with many fluorescent sensors, because of its paramagnetic nature [14,15,16]. In our previous study, we synthesized four types of 3-[bis(pyridin-2-ylmethyl)amino]-BODIPYs with bis(pyridin-2-ylmethyl)amine [di(2-picolyl)amine] as the cation receptor at the 3-position of BODIPY [17]. We found that substitution with the electron-withdrawing sulfonylphenyl group at the 5-position gives the highest fluorescence quantum yield and largest absorption coefficient in the presence of Cu^2+^. In this study, we introduced another type of electron-withdrawing group (i.e., a carboxyphenyl group) at the 5- position of 3-[bis(pyridin-2-ylmethyl)amino]-BODIPY, and evaluated its photophysical properties and Cu^2+^-sensing ability.

## 2. Materials and Methods

### 2.1. Synthesis of BODIPY ***1***

**Figure 1:**
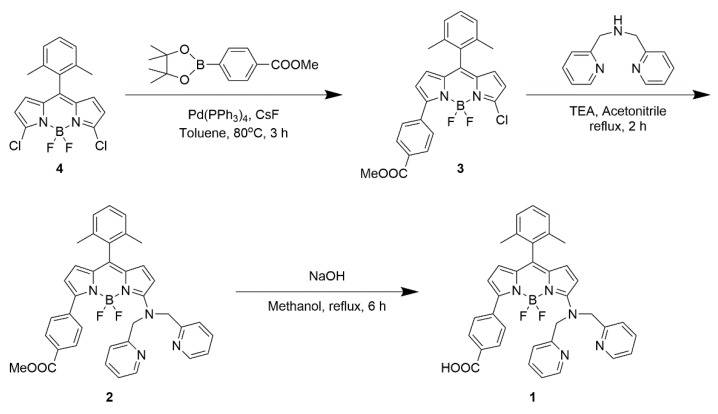
Synthetic route to BODIPY **1**.

Unless otherwise stated, all reagents were purchased from Sigma-Aldrich (St. Louis, MO, USA), Wako Pure Chemical Industries (Osaka, Japan), or Tokyo Chemical Industry (Tokyo, Japan), and used without further purification. Figure 1 shows the synthetic route to BODIPY **1**. High-performance thin-layer chromatography (HPTLC; silica gel 60 F_254_, Merck KGaA, Darmstadt, Germany) or thin-layer chromatography (aluminum oxide 60 F_254_, basic, Merck KGaA, Darmstadt, Germany) were used to monitor the reactions. HPTLC plates were visualized under ultraviolet light and/or by staining with anisaldehyde solution (anisaldehyde/ethanol/sulfuric acid/acetic acid = 1.9/68/2.5/1.2, *v/v*), followed by heating for a few minutes. Silica gel 60 (230–400 mesh) or aluminum oxide 90 active basic (Merck KGaA, Darmstadt, Germany) were used for open-column chromatography. ^1^H NMR spectra were recorded with a JEOL 400 (400 MHz) spectrometer (JEOL Ltd., Tokyo, Japan) at room temperature. Chemical shifts in the NMR spectra are reported in parts per million, relative to tetramethylsilane as the internal standard (residual CHCl_3_; 7.26 ppm). Coupling constants (*J*) are reported in hertz. Splitting patterns are indicated as s, singlet; d, doublet; t, triplet; q, quartet; m, multiplet; brs, broad singlet for ^1^H NMR data. High-resolution mass spectroscopy (HRMS) was performed with a Thermo Scientific Exactive (Thermo Fisher Scientific K.K., Tokyo, Japan) or JEOL JMS-T100GCv mass spectrometer (JEOL Ltd., Tokyo, Japan).

#### 2.1.1. 3-Chloro-4,4-difluoro-5-[4-(methoxycarbonyl)phenyl]-8-(2,6-dimethylphenyl)-4-bora-3a,4a-diaza-*s*-indacene (**3**)

Compound **4** was synthesized according to the previously reported method [17]. Compound **4** (120 mg, 0.33 mmol, 1 equiv), 2-(4-methoxycarbonylphenyl)-4,4,5,5-tetramethyl-1,3,2-dioxaborolane (86 mg, 0.33 mmol, 1 equiv), cesium fluoride (150 mg, 0.99 mmol, 3 equiv), and tetrakis(triphenylphosphine)palladium (5 mol %) were dissolved in dry toluene. The reaction mixture was stirred at 80 °C for 3 h under nitrogen. The mixture was cooled to room temperature, water was added, and the organic layer was extracted with dichloromethane. The combined organic layers were washed with brine, and then dried over Na_2_SO_4_. The solvent was evaporated and the crude product was purified by column chromatography on silica gel (hexane/ethyl acetate = 8/1) to yield compound **3** (38%) as a dark-red solid. ^1^H NMR (400 MHz, CDCl_3_, Figure S1): *δ* = 8.15 (d, 2H, *J* = 8.4 Hz), 8.03 (d, 2H, *J* = 8.4 Hz), 7.31 (t, 1H, *J* = 7.6 Hz), 7.16 (d, 2H, *J* = 7.6 Hz), 6.70 (d, 1H, *J* = 4.3 Hz), 6.65 (d, 1H, *J* = 4.2 Hz), 6.59 (d, 1H, *J* = 4.2 Hz), 6.36 (d, 1H, *J* = 4.2 Hz), 3.95 (s, 3H), 2.19 (s, 6H). HRMS (ESI, Figure S2) *m/z* calcd for [M *+* Na]^+^ C_25_H_20_BClF_2_N_2_O_2_Na 486.1203; found 486.1207. 

#### 2.1.2. 3-[Bis(pyridin-2-ylmethyl)amino]-4,4-difluoro-5-[4-(methoxycarbonyl)phenyl]-8-(2,6-dimethylphenyl)-4-bora-3a,4a-diaza-*s*-indacene (**2**)

Compound **3** (63 mg, 0.14 mmol, 1 equiv) was dissolved in acetonitrile. Di(2-picolyl)amine (41 mg, 0.20 mmol, 1.5 equiv) and triethylamine (0.57 mL, 4.07 mmol, 30 equiv) were then added to the solution. The reaction mixture was refluxed with stirring for 2 h under nitrogen. The mixture was cooled to room temperature, ethyl acetate was added, and the mixture was washed with water and brine. The organic layer was dried over Na_2_SO_4_, and the solvent was evaporated. The crude product was purified by column chromatography on aluminum oxide basic (hexane/ethyl acetate = 1/1) to yield compound **2** (76%) as a dark-red solid. ^1^H NMR (400 MHz, CDCl_3_, Figure S3): *δ* = 8.53 (d, 2H, *J* = 4.8 Hz), 8.02 (d, 2H, *J* = 8.2 Hz), 7.92 (d, 2H, *J* = 8.3 Hz), 7.64 (td, 2H, *J* = 7.7, 1.7 Hz), 7.34 (d, 2H, *J* = 7.8 Hz), 7.23 (t, 1H, *J* = 7.6 Hz), 7.19–7.16 (m, 2H), 7.10 (d, 2H, *J* = 7.6 Hz), 6.55 (d, 1H, *J* = 5.1 Hz), 6.44 (d, 1H, *J* = 3.8 Hz), 6.24 (d, 1H, *J* = 5.1 Hz), 6.16 (d, 1H, *J* = 3.8 Hz), 5.20 (s, 4H), 3.91 (s, 3H), 2.18 (s, 6H). HRMS (ESI, Figure S4) *m/z* calcd for [M *+* Na]^+^ C_37_H_32_BF_2_N_5_O_2_Na 649.2546; found 649.2553.

#### 2.1.3. 3-[Bis(pyridin-2-ylmethyl)amino]-4,4-difluoro-5-(4-carboxyphenyl)-8-(2,6-dimethylphenyl)-4-bora-3a,4a-diaza-*s*-indacene (**1**)

Compound **2** (37 mg, 0.06 mmol) was dissolved in methanol (15 mL). An aqueous solution of sodium hydroxide (0.2 M, 1.5 mL) was then added to the solution. The reaction mixture was refluxed with stirring for 6 h. The mixture was cooled to room temperature, and then the pH was adjusted to between 4 and 5, by adding an aqueous solution of hydrochloric acid (2 M). Ethyl acetate was added and the mixture was washed with water and brine. The organic layer was dried over Na_2_SO_4_ and the solvent was evaporated to yield BODIPY **1** (63%) as a dark-red solid. ^1^H NMR (400 MHz, CDCl_3_, Figure S5): *δ* = 8.54 (d, 2H, *J* = 4.8 Hz), 8.06 (d, 2H, *J* = 8.4 Hz), 7.94 (d, 2H, *J* = 8.4 Hz), 7.64 (td, 2H, *J* = 7.7, 1.7 Hz), 7.37 (d, 2H, *J* = 8.0 Hz), 7.23 (t, 1H, *J* = 7.6 Hz), 7.19–7.16 (m, 2H), 7.10 (d, 2H, *J* = 7.6 Hz), 6.95 (d, 1H, *J* = 3.0 Hz), 6.56 (d, 1H, *J* = 5.1 Hz), 6.45 (d, 1H, *J* = 3.8 Hz), 6.28 (d, 1H, *J* = 5.1 Hz), 6.17 (d, 1H, *J* = 3.8 Hz), 5.23 (s, 4H), 2.18 (s, 6H). HRMS (ESI, Figure S6) *m/z* calcd for [M *+* H]^+^ C_36_H_31_BF_2_N_5_O_2_H 613.2570; found 613.2574.

### 2.2. Fluorescence and UV–Vis Spectroscopic Measurements

Fluorescence spectra were recorded with a Hitachi F-7100 fluorescence spectrophotometer (Hitachi High-Technologies Corporation, Tokyo, Japan) and absorption spectra were recorded with a Shimadzu UV-1800 spectrophotometer (SHIMADZU CORPORATION, Kyoto, Japan). Analytical grade acetonitrile was used for all spectroscopic experiments. Stock solution of BODIPY **1** (40 μM) was prepared by dissolving BODIPY **1** in acetonitrile, and stock solutions of cations (300 μM) were prepared by dissolving metallic perchlorate in acetonitrile. Each test sample was prepared by adding an aliquot of the BODIPY **1** and cation stock solutions to a 10 mL volumetric flask, and then diluting the sample with acetonitrile. For measurements of absorption spectra, an appropriate volume of the cation stock solution was added to a reference cell to ensure absorption spectrum of the test solution. The excitation and emission slit widths were both 5.0 nm. The fluorescence quantum yields of the test solution were calculated by comparing the area under the corrected fluorescence spectrum of the test solution with that of an ethanol solution of rhodamine 6G, which has a fluorescence quantum yield of 0.95 [18]. The fluorescence quantum yield of each sample was obtained as

*Ф*_S_ = *Ф*_R_ × *S*_S_/*S*_R_ × *A*_R_/_AS_ × (*η*_S_/*η*_R_)^2^, (1)

where *Ф* is the fluorescence quantum yield, *S* is the integrated area of the fluorescence spectrum, *A* is the absorbance at the excitation wavelength, *η* is the refractive index of the solvent, and the subscripts, *S* and *R*, refer to the sample and the reference fluorescent dye (i.e., rhodamine 6G), respectively. All spectroscopic measurements were conducted in at least duplicate.

## 3. Results and Discussion

### 3.1. Photophysical Properties of BODIPY ***1***

Table 1 shows the photophysical properties of BODIPY **1**. BODIPY **1** showed a broad absorption band at around 503 nm, with a large molar absorption coefficient (*ε*_503_ = 25,000 M^−1^ cm^−1^); it also showed a fluorescence peak at 580 nm. The fluorescence quantum yield and the Stokes shift were 0.34 and 2600 cm^−1^, respectively. The absorption and fluorescence maximum wavelengths, molar absorption coefficient, and Stokes shift of BODIPY **1** were comparable to those of a previously reported sulfonylphenyl-substituted BODIPY cation sensor [17]. However, the fluorescence quantum yield of BODIPY **1** was lower than that of the sulfonylphenyl-substituted BODIPY.

### 3.2. Spectroscopic Response of BODIPY ***1*** toward Metal Cations

**Figure 2:**
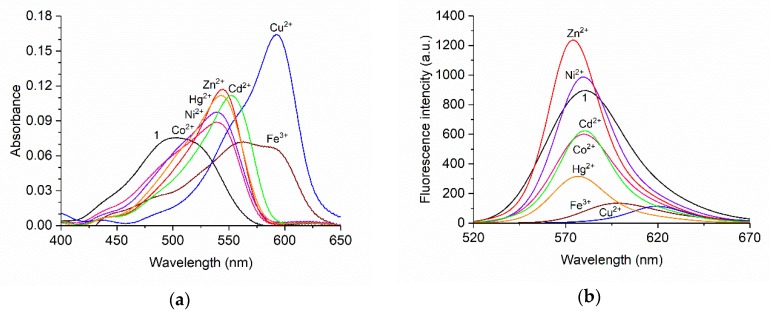
(**a**) Absorption and (**b**) fluorescence spectra of BODIPY **1** (3 µM) in the presence of different metal cations (300 µM) in acetonitrile with excitation at 510 nm.

**Figure 3:**
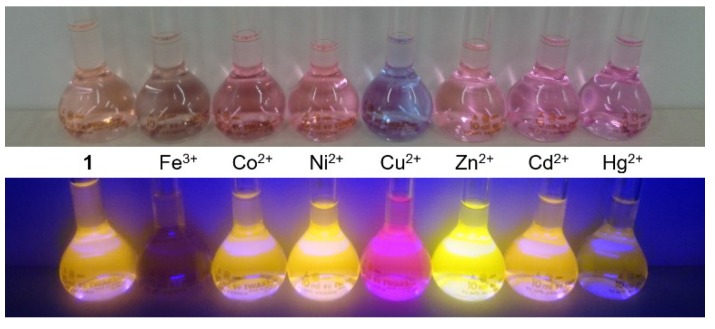
Photos of BODIPY **1** solution (3 µM) in the presence of different metal cations (300 µM) in acetonitrile. The fluorescence color was observed under excitation at 365 nm.

Fluorescence and absorption spectroscopic response of BODIPY **1** was investigated with different metal cations to clarify its cation-sensing ability. Figure 2 shows the absorption and fluorescence spectra of BODIPY **1** with and without the respective metal cations (100 equiv). The absorption spectra of BODIPY **1** showed a broad absorption band at around 503 nm. The addition of metal cations caused the spectra to red-shift. The largest red-shift, to 593 nm, was observed on addition of Cu^2+^. Under excitation at 510 nm, Zn^2+^ and Ni^2+^ enhanced the fluorescence intensity of BODIPY **1,** and the fluorescence peak was slightly blue-shifted from 580 nm to 574 nm and 579 nm for Zn^2+^ and Ni^2+^, respectively. In contrast, Co^2+^, Cd^2+^, and Hg^2+^ weakened the fluorescence intensities of BODIPY **1**. Fe^3+^ and Cu^2+^ induced red-shifts of the fluorescence band of BODIPY **1**. In particular, the fluorescence peak of BODIPY **1** at 580 nm underwent a large red-shift to 620 nm on addition of Cu^2+^. Figure 3 is the photos of BODIPY **1** solution with and without the respective metal cations (100 equiv). The changes in absorption and fluorescence color caused by Cu^2+^ addition were easily distinguishable by the naked eye. Based on these results, we expected the Cu^2+^ selectivity of BODIPY **1**, because Cu^2+^ induced the largest red-shifts of the fluorescence and the absorption spectra of BODIPY **1**. Therefore, the Cu^2+^-sensing ability of BODIPY **1** was investigated in the next step. The fluorescence spectral shift enables ratiometric measurement of Cu^2+^ concentrations.

### 3.3. Ratiometric Determination of Cu^2+^ with BODIPY ***1***

**Figure 4:**
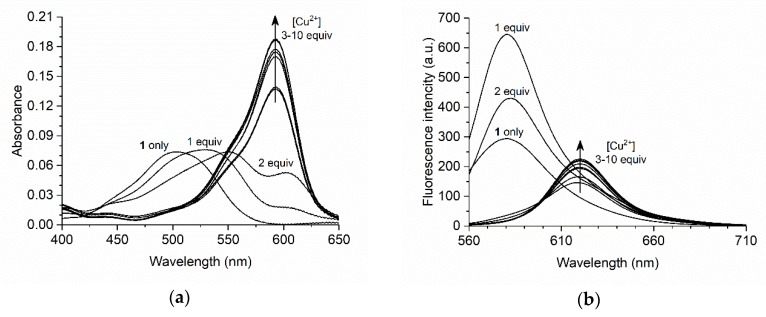
Changes in (**a**) absorption and (**b**) fluorescence spectra of BODIPY **1** (3 µM) with increasing Cu^2+^ concentration (0–30 µM) in acetonitrile. The excitation wavelength was 550 nm.

Table 2 shows the photophysical properties of BODIPY **1** in the presence of Cu^2+^ (10 equiv). On addition of Cu^2+^, the absorption peak red-shifted from 503 nm to 593 nm and the fluorescence peak red-shifted from 580 nm to 620 nm. Such red-shifted spectra were also observed for previously reported BODIPY sensors with structural similarities to BODIPY **1** [10,12]. The fluorescence quantum yield decreased from 0.34 to 0.07. The absorption and fluorescence spectra of BODIPY **1** with a variety of Cu^2+^ concentrations in acetonitrile are shown in Figure 4. In the absorption spectra, the absorption peak was red-shifted by the addition of 1 and 2 equiv of Cu^2+^. On addition of 3 equiv of Cu^2+^, a new absorption peak appeared at 593 nm, and its intensity gradually increased with increasing Cu^2+^ concentration. In the fluorescence spectra, the intensity of the fluorescence band at around 580 nm increased on addition of 1 and 2 equiv of Cu^2+^. On addition of 3 equiv of Cu^2+^, a new fluorescence peak appeared at 620 nm, and its intensity gradually increased with increasing concentration of Cu^2+^. Absorption and fluorescence spectral changes almost stopped on addition of 10 equiv of Cu^2+^. These results indicate that the stoichiometry of the BODIPY **1**/Cu^2+^ complex changes during the addition of 1–3 equiv of Cu^2+^ (i.e., 1:1, 1:2, and 1:3 complex of BODIPY **1**/Cu^2+^) because more than two absorption peaks were observed. The absorption peak at 593 nm and the fluorescence peak at 620 nm might originate from 1:3 complex of BODIPY **1** and Cu^2+^, because the distinctive spectral changes were observed upon addition of 3 equiv of Cu^2+^. Therefore, we next investigated the stoichiometry of the system.

**Figure 5:**
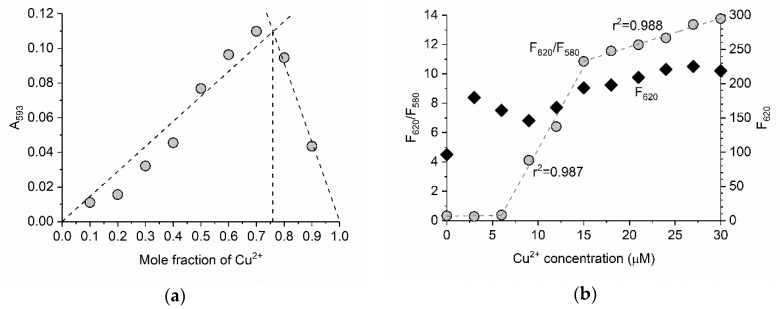
(**a**) Job’s plot of BODIPY **1** and Cu^2+^ system. The total concentration of BODIPY **1** and Cu^2+^ was 9 µM and absorbance at 593 nm (A_580_) was plotted. (**b**) Plot of fluorescence intensity at 620 nm (*F*_620_) and ratio of fluorescence intensities (*F*_620_/*F*_580_) of BODIPY **1** (3 µM) versus increasing Cu^2+^ concentration (0–30 µM) in acetonitrile. The excitation wavelength was 550 nm.

To investigate the binding stoichiometry of BODIPY **1** and Cu^2+^, the Job’s method was used [19]. Figure 5a shows the Job’s plot of the absorbance at 593 nm (*A*_593_). *A*_593_ was highest when the mole fraction of Cu^2+^ was 0.7. The intersection of the fitted lines occurred at approximately 0.75 of the mole fraction of Cu^2+^, suggesting a 1:3 stoichiometry of BODIPY **1** and Cu^2+^. Although we did not introduce the carboxyphenyl group as a cation receptor, the group and acetonitrile might participate in the binding event. The ratio of the fluorescence intensity at 620 nm to that at 580 nm (*F*_620_/*F*_580_) was calculated for ratiometric determination of Cu^2+^ (Figure 5b). The *F*_620_/*F*_580_ value was unchanged in the range 0–6 µM Cu^2+^ (i.e., 0–2 equiv). In the range 6–15 µM Cu^2+^, *F*_620_/*F*_580_ increased linearly from 0.4 to 10.8. Further addition of Cu^2+^ led to a gradual increase in *F*_620_/*F*_580_, and the slope of the plot became low. These results indicate that Cu^2+^ concentration can be determined in the range 6–30 µM, and the limit of quantification for Cu^2+^ was 6 µM. On the other hand, *F*_620_ did not linearly increase depending on the Cu^2+^ concentration, which suggests the ratiometric measurement is preferable. To the best of our knowledge, BODIPY **1** is the third example of a ratiometric fluorescent sensor for Cu^2+^ based on BODIPY [17,20]. Compared to these sensors, BODIPY **1** shows better quantitative performance for Cu^2+^ because the linearity of the calibration curve is well established.

**Figure 6:**
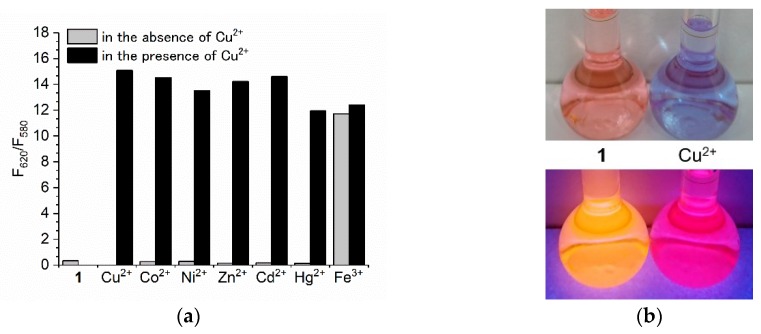
(**a**) Changes in the ratio of fluorescence intensities (*F*_620_/*F*_580_) of BODIPY **1** (3 µM) on addition of different metal cations. The gray bars represent the addition of each cation (30 µM). The black bars represent subsequent addition of Cu^2+^ (30 µM). The excitation wavelength was 550 nm. (**b**) Photos of BODIPY **1** solution (4 µM) containing tap water (acetonitrile/tap water = 9/1, *v/v*) in the absence (**1**, left flask) and the presence of Cu^2+^ (Cu^2+^, right flask). Cu^2+^ was spiked with tap water in advance. The concentration of Cu^2+^ in tap water was 30 ppm (470 µM). The fluorescence color was observed under excitation at 365 nm.

The selectivity of BODIPY **1** for Cu^2+^ was investigated (Figure 6a). Co^2+^, Ni^2+^, Zn^2+^, Cd^2+^, and Hg^2+^ did not increase the fluorescence intensity at 620 nm, which originated from the complex of BODIPY **1** with Cu^2+^. Addition of the cations, therefore, did not cause *F*_620_/*F*_580_ to increase. However, when Cu^2+^ was added to these solutions, *F*_620_ increased and therefore *F*_620_/*F*_580_ increased. This Cu^2+^ selectivity might be a result of the interaction of BODIPY-bis(pyridin-2-ylmethyl)amine conjugate and Cu^2+^, which has a low-lying *d–d* state nature [10]. Among these cations, Fe^3+^ is the main interfering cation in the use of BODIPY **1** as a Cu^2+^ fluorescent sensor, because the fluorescence spectrum of the solution containing 10 equiv of Fe^3+^ overlaps with that of the solution containing Cu^2+^. The absorption spectra shown in Figure 2a also suggest the formation of the complex of BODIPY **1** and Fe^3+^. Therefore, some kind of pretreatment for Fe^3+^ removal would be necessary for Cu^2+^ sensing with BODIPY **1**. Fe^3+^ also has a paramagnetic nature, and interfering effects for Cu^2+^ sensors were often reported [21,22,23]. To investigate the possibility of practical application for Cu^2+^ screening with BODIPY **1**, we used tap water (pH = 7.4, electrical conductivity=19.7 mS/m) as a real sample. Cu^2+^ was spiked with tap water, and the concentration of Cu^2+^ was adjusted to 30 ppm (470 µM), which is ten-times higher than the national effluent standards for Cu in Japan. Figure 6b shows the photos of BODIPY **1** solution containing 10% of prepared tap water. The absorption color changed from light pink to light purple by the addition of Cu^2+^. On the other hand, the fluorescence color changed from orange to red purple. Both color changes were easily distinguishable by the naked eye, which is valuable for on-site screening of Cu^2+^.

## 4. Conclusions

In this study, we synthesized BODIPY **1** as a ratiometric fluorescent sensor for Cu^2+^. BODIPY **1** showed large absorption and fluorescence spectral red-shifts on binding to Cu^2+^. The fluorescence quantum yield of the complex was 0.7. The Job’s plot revealed 1:3 stoichiometry of BODIPY **1** and Cu^2+^. The ratio of the fluorescence intensity (*F*_620_/*F*_580_) increased with increasing concentration of Cu^2+^ (6–30 µM). Fe^3+^ was the main interfering cation for Cu^2+^ sensing with BODIPY **1**. In addition, BODIPY **1** could be used for Cu^2+^ screening in a water-containing sample. The change in fluorescence color by Cu^2+^ could be easily recognized by the naked eye.

## Figures and Tables

**Table 1:** Photophysical properties of BODIPY **1** in acetonitrile.

*λ*_abs_	*λ*_flu_	*ε*_503_	*Ф*	Stokes Shift
(nm)	(nm)	(M^−1^ cm^−1^)	(-)	(cm^−1^)
503	580	25,000	0.34	2600

**Table 2:** Photophysical properties of BODIPY **1** with Cu^2+^ (10 equiv) in acetonitrile.

*λ*_abs_	*λ*_flu_	*ε*_593_	*Ф*
(nm)	(nm)	(M^−1^ cm^−1^)	(-)
593	620	59,000	0.07

## Supplementary Materials

The following are available online at http://www.mdpi.com/1996-1944/11/5/814/s1, Figure S1: ^1^H NMR spectrum of compound **3**, Figure S2: HRMS spectrum of compound **3**, Figure S3: ^1^H NMR spectrum of compound **2**, Figure S4: HRMS spectrum of compound **2**, Figure S5: ^1^H NMR spectrum of BODIPY **1**, Figure S6: HRMS spectrum of BODIPY **1**.
