# Supplementary material for: 3-[Bis(pyridin-2-ylmethyl)amino]-5-(4-carboxyphenyl)-BODIPY as Ratiometric Fluorescent Sensor for Cu2+
Source: Materials (Basel). 2018 May 16;11(5):814. doi: 10.3390/ma11050814 (PMC5978191; doi:10.3390/ma11050814)
Supplement: Supplementary file 1 [file materials-11-00814-s001.pdf]

Supplementary Materials

3-[Bis(pyridin-2-ylmethyl)amino]-5-(4-carboxyphenyl)-BODIPY as Ratiometric Fluorescent Sensor for Cu^{2+}

Akira Hafuka [1,*], Hisashi Satoh [2], Koji Yamada [3], Masahiro Takahashi [2] and Satoshi Okabe [2]

[1] Department of Integrated Science and Engineering for Sustainable Society, Faculty of Science and Engineering, Chuo University, 1-13-27 Kasuga, Bunkyo-ku, Tokyo 112-8551, Japan

[2] Division of Environmental Engineering, Faculty of Engineering, Hokkaido University, North-13, West-8, Sapporo 060-8628, Japan; qsatoh@eng.hokudai.ac.jp (H.S.); m-takaha@eng.hokudai.ac.jp (M.T.); sokabe@eng.hokudai.ac.jp (S.O.)

[3] Division of Environmental Materials Science, Graduate School of Environmental Science, Hokkaido University, North-10, West-5, Sapporo 060-0810, Japan; yamada@ees.hokudai.ac.jp

* Correspondence: hafuka.14p@g.chuo-u.ac.jp; Tel.: +81-3-3817-7283

Received: 8 April 2018 Accepted: 14 May 2018; Published: date

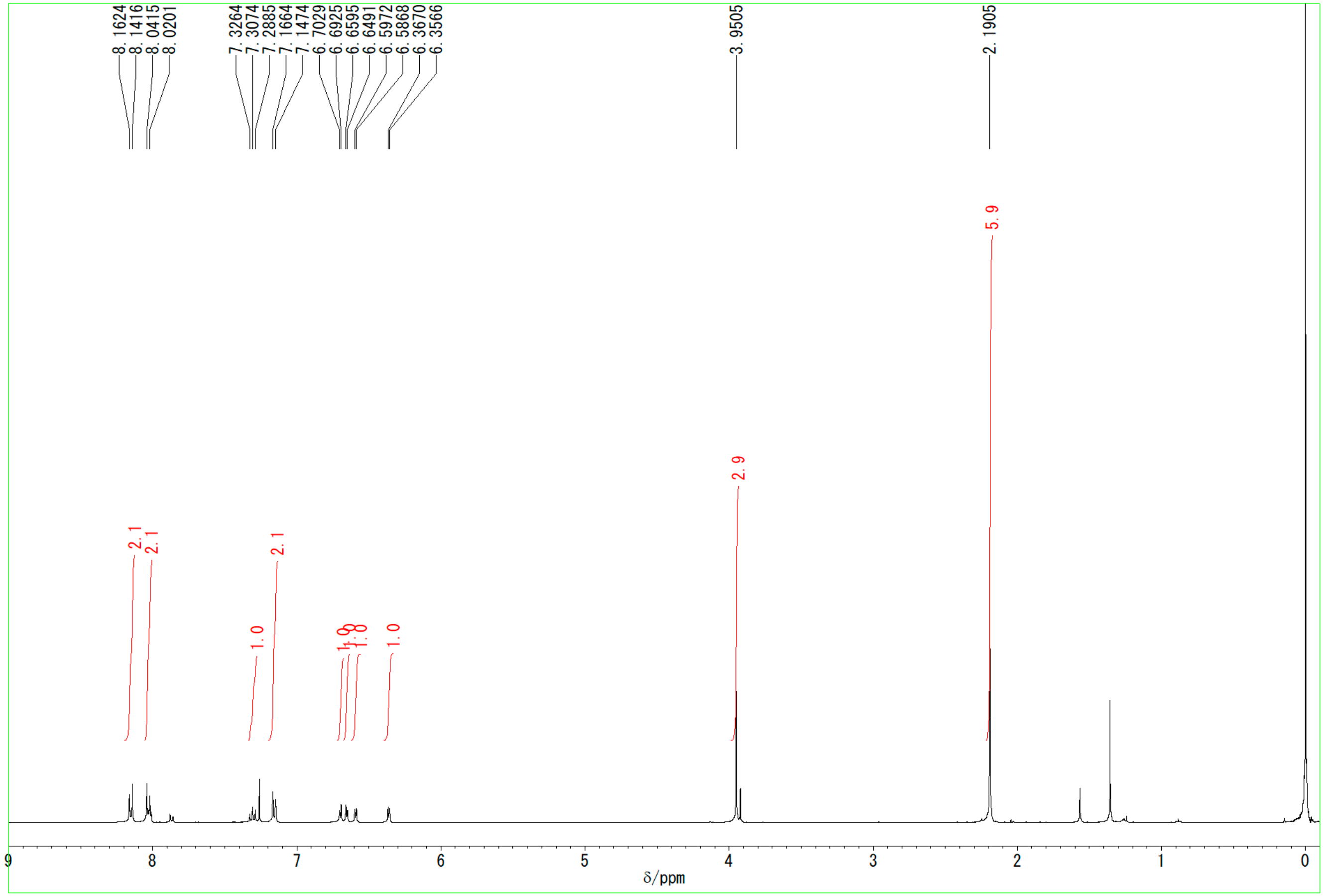

Figure S1. ^{1}H NMR spectrum of compound **3**.

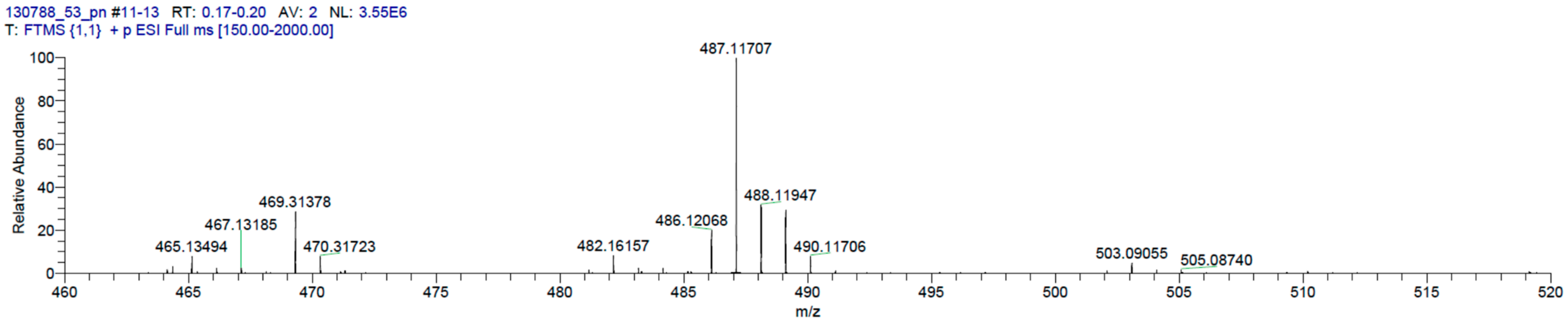

Figure S2. HRMS spectrum of compound **3**.

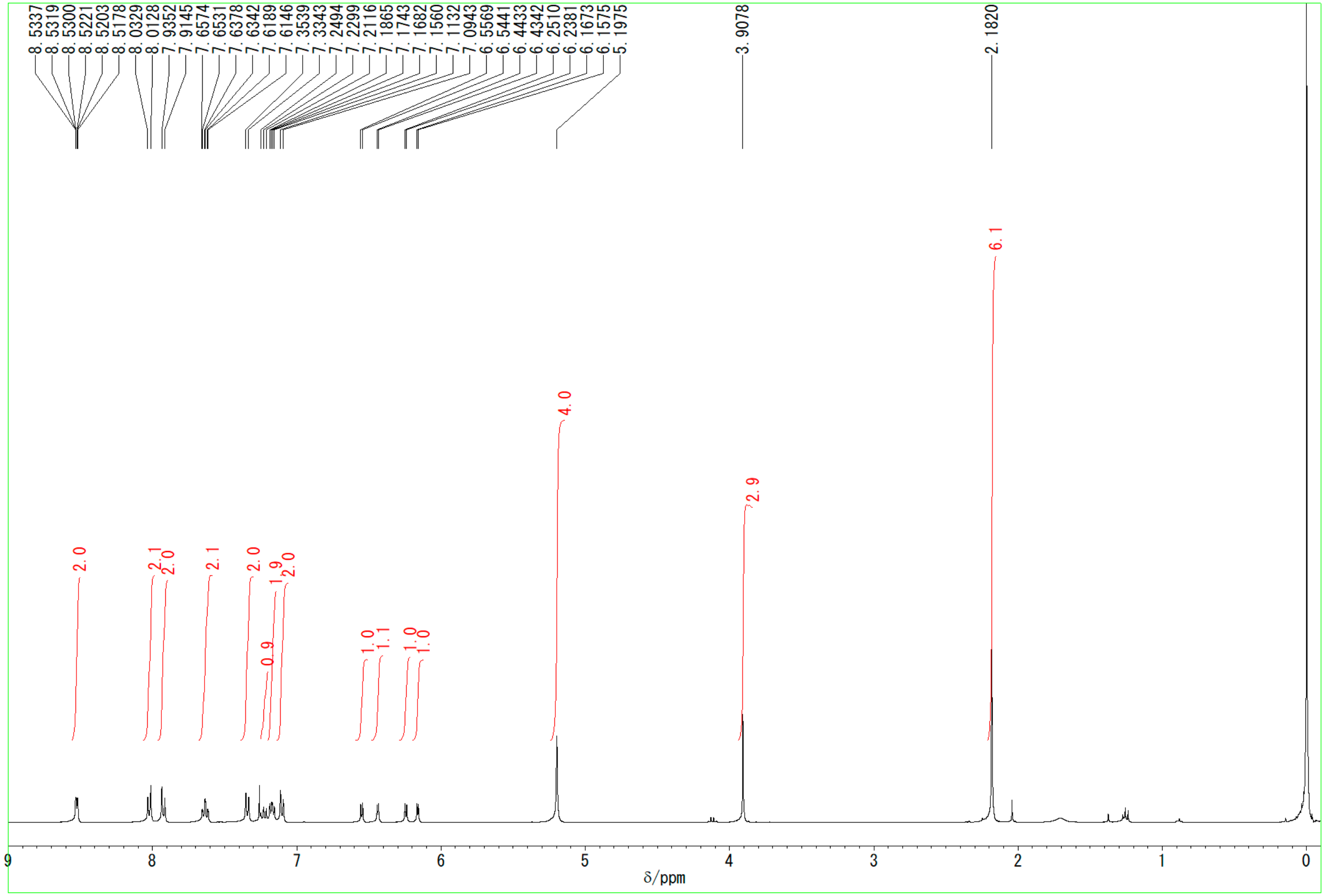

Figure S3. ^{1}H NMR spectrum of compound **2**.

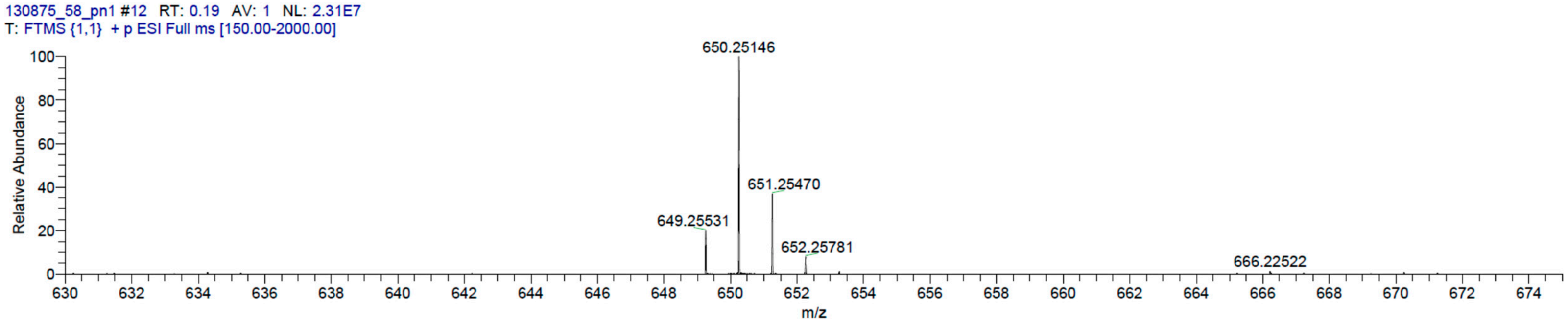

Figure S4. HRMS spectrum of compound **2**.

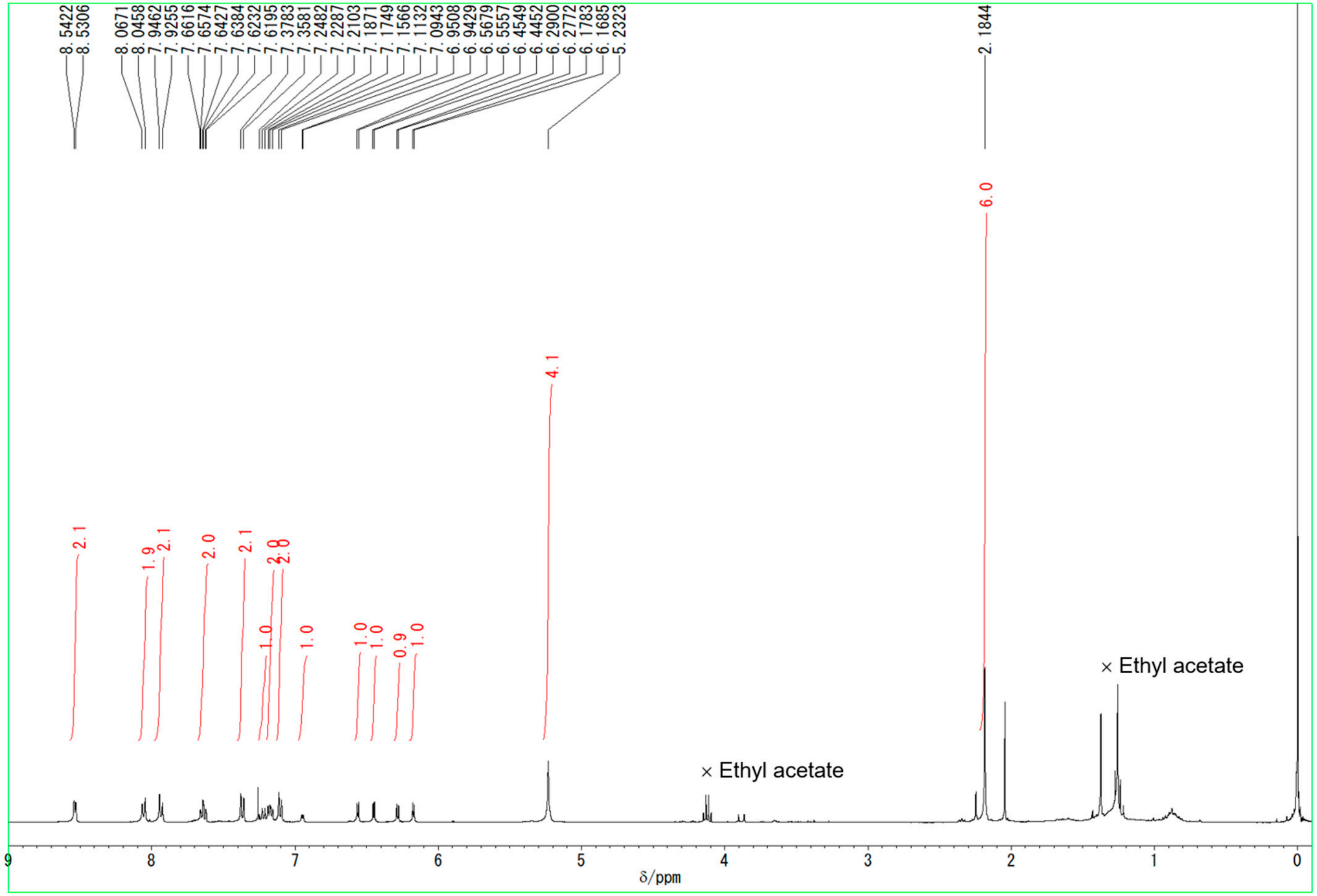

Figure S5. ^{1}H NMR spectrum of BODIPY **1**.

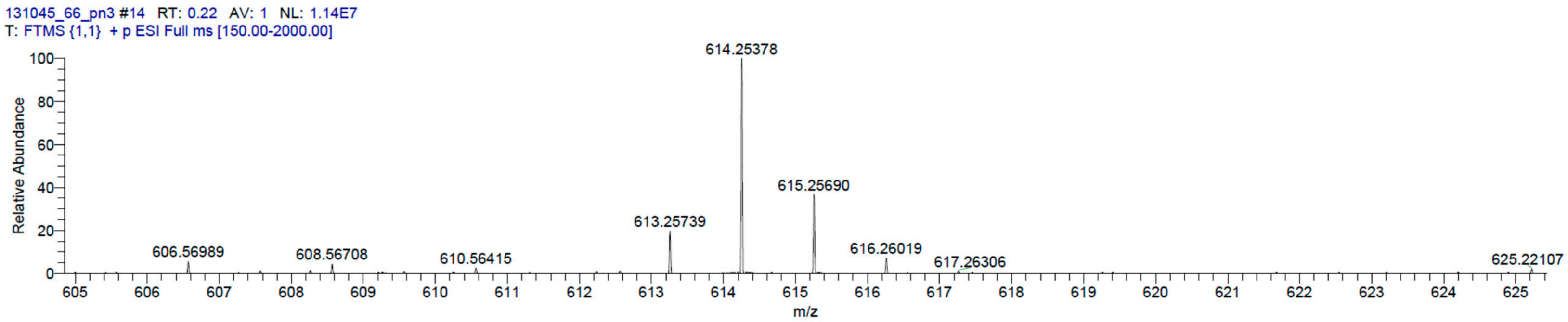

Figure S6. HRMS spectrum of BODIPY **1**.
